# Bone marrow micrometastasis in breast cancer: review of detection methods, prognostic impact and biological issues

**DOI:** 10.1136/jcp.2007.046649

**Published:** 2007-11-23

**Authors:** A Vincent-Salomon, F C Bidard, J Y Pierga

**Affiliations:** Institut Curie, Paris, France

## Abstract

Immunocytochemical detection of disseminated tumour cells in the bone marrow of patients with primary breast cancer at surgery has been shown to be an independent prognostic factor in single institutional studies and in a large pooled analysis. However, bone marrow sampling and assessment of disseminated tumour cells is not a routine procedure in the clinical management of patients with breast cancer, but will certainly play a role in the near future for risk stratification and monitoring of therapeutic efficacy. Accurate identification of disseminated tumour cells in bone marrow must be based on standardised methodologies and procedures. This review describes these methodologies and the standardised morphological criteria used for disseminated tumour cell detection. The prognostic value of circulating tumour cells detection in peripheral blood is demonstrated in patients with metastatic disease but remains to be substantiated at early stage. The significance of disseminated tumour cells in bone marrow and in the blood for the prediction of response to therapy is briefly summarised. Finally, this review addresses the main biological questions raised by disseminated tumour cells, in particular understanding tumour dormancy and identifying metastatic stem cells.

In clinical practice, the most important prognostic information about breast cancer is provided by pathological staging, such as tumour grade, tumour size, presence of lymphatic and vascular invasion, axillary lymph node involvement and steroid receptor status. Nonetheless, about 20–30% of patients with a favourable prognosis relapse within 5 years and many patients with poor prognostic factors will survive for more than 10 years. In this context, there is a real need for new, more accurate prognostic factors. One of the promising new parameters is identification of the presence of disseminated tumour cells (DTC) in bone marrow (BM). DTC, the most precise term, are also described by several synonyms such as bone marrow micrometastasis or minimal residual disease. The presence of BM DTC is clearly associated with a poor outcome for patients with stage I to III breast cancer.^1^ The procedure is still investigational according to the American Society of Clinical Oncology 2007 update of recommendations for the use of tumour markers in breast cancer,^2^ and its incorporation into clinical management algorithms is currently the focus of research. Many different methodologies have been used to detect DTC, but standardised guidelines have now been published.^3^ The current challenge for pathologists is to improve and standardise early detection of DTC. In this review, we will summarise the methodologies most commonly used to detect DTC, discuss the clinical impact of DTC in bone marrow at initial diagnosis and during follow-up and treatment evaluation, and highlight the biological and clinical questions raised by DTC.

## METHODOLOGIES FOR DETECTION OF BONE MARROW MICROMETASTASES

The methodology most commonly used to detect DTC is immunocytochemistry performed on BM aspirates. Immunocytochemistry currently remains the gold standard for BM DTC detection, with a sensitivity ranging from 1 DTC in 10^5^ to 1 in 10^6^ leucocytes.

### Bone marrow aspiration

Ideally, this procedure should be performed under general anaesthesia, at the time of initial surgery, before the skin incision. If necessary, it can be performed under local anaesthesia. Bone marrow aspirates are usually performed from both anterior iliac crests, as no difference has been reported between anterior and posterior iliac crest aspirations.^4^ Bone marrow (5–10 ml) should be aspirated and pooled in heparinised tubes, EDTA or sodium citrate until further processing. Optimal storage temperature is at 4–25°C. A Ficoll density gradient centrifugation for tumour cell enrichment is performed, ideally within the first 24 hours after collection. A cell count is performed on the interphase layer containing mononuclear cells, and cytospins are prepared and smeared on positively charged glass slides; 2–3×10^6^ cells per patient are examined. The slides (3–6 slides per patient) are air-dried at 4°C or at room temperature overnight before fixation^3^ and immunostaining.

### Immunocytostaining

#### Antibodies

The majority of studies use the fact that breast cancer is an epithelial cell tumour and that BM normally does not contain any epithelial cells. Various antibodies have been used over the years: initially polyclonal antibodies raised against epithelial membrane antigen (EMA), which was subsequently abandoned as this antibody can cross-react with plasma cells and immature precursors in bone marrow; then monoclonal antibodies raised against various cytokeratins, mucins (MUC1), mammaglobin and adhesion molecules such as EpCAM. The most commonly used antibody at the present time is A45/BB3 (Micromet, Munich, Germany), a monoclonal antibody that reacts with common epitopes on several cytokeratins including CK8, CK18 and CK19. Other antibodies include CK2 (mouse IgG1, Boehringer, Mannheim) directed against CK18, and AE1/AE3 that reacts with basic and acidic keratins covering a large spectrum of cytokeratins (CK10, CK14–16, CK19 and CK1-8). The recommended revelation system is the alkaline–anti-alkaline phosphatase technique with levamisole as blocking agent. Cells are counterstained with haematoxylin to visualise their nuclear morphology.

#### Quality control

Because haematopoietic cells can sometimes be stained by anti-cytokeratin antibodies,^5^ rigorous internal and external quality control procedures must be applied.

##### Internal controls

Before the technique can be used in clinical practice, it should first be evaluated on bone marrow samples from patients without cancer (orthopaedic surgery specimens, for example) in order to validate its specificity. The technique must also be calibrated (primary antibodies and revelation system dilutions) using breast cancer cell lines such as MCF7 or SKBR3 at different dilutions, spiked into mononuclear cells from patients without breast cancer. All specimens from breast cancer patients must also be systematically examined in parallel with controls, consisting of slides stained with isotype-matched immunoglobulin.

##### External controls

Ring experiments are highly recommended to improve the between-centre reproducibility of bone marrow analysis.

### Analysis of cell preparations

Morphological analysis has been clearly shown to improve the specificity of DTC identification and is highly recommended, but optimal separation of DTC from cytokeratin-positive haematopoietic or non-haematopoietic cells remains challenging, as it is often difficult to detect single DTC in mononuclear cell fractions from BM. Manual screening of 2–3×10^6 ^cells and 2–3×10^6 ^negative control cells using light microscopy is performed by an experienced observer. Morphological analysis of cytokeratin-positive cells is based on consensus criteria.^3^ ^6^ The read-out of positive cells should be controlled by at least two independent observers. The screening of large volumes of material by immunocytochemical techniques can be time-consuming; automated image-analysis systems can be used. In a European interlaboratory testing of well-known procedures for immunocytochemical detection of epithelial cells in bone marrow, the MDS1 from Applied Imaging screening sensitivity was similar to manual screening, while ACIS from Chromavision detected fewer cells.^7^

Standardised interpretation according to European guidelines^3^ ^6^ ^7^ is required to improve the specificity of DTC detection.

- Samples are classified into two categories: positive or negative.
-
  - Positive samples are those with cytokeratin-positive cells with disseminated tumour cell morphology. The number of cells should be indicated.
  - Negative samples are those with no positive immunocytochemical stained cells or cytokeratin-positive cells without disseminated tumour cell morphology (e.g. haematopoietic cells, squamous cells).
- All cytokeratin-positive cells should be classified as disseminated tumour cells, i.e. cytokeratin-positive cells with disseminated tumour cell morphology or cytokeratin-positive cells.

**Figure 1:**
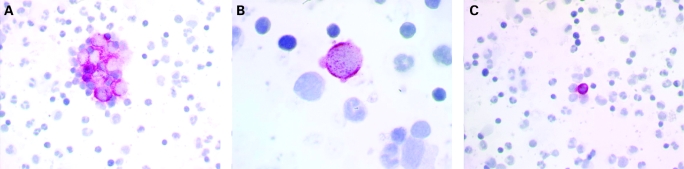
Examples of A45/BB3-positive disseminated tumour cells (DTC) and haematopoietic cells. (A) Clusters of DTC. (B) Isolated DTC. The cell is taller than other surrounding cells, with a high nuclear/cytoplasmic ratio; it shows strong and irregular cytoplasmic staining for cytokeratin. (C) Haematopoietic cell.

The morphological features of DTC are:

- The presence of cell clusters (fig 1A).
- Large cell size with a clearly enlarged nuclear size and a high nuclear-to-cytoplasmic ratio (fig 1B), and strong or irregular cytoplasmic staining for cytokeratin.
- Cytokeratin filaments can be seen.
- Staining partially covers the nucleus. A large nucleolus can be seen and the nucleus is often granular or stippled (fig 1B).

Some cytokeratin cells are clearly recognised as haematopoietic or squamous cells (fig 1C).

According to morphological classification guidelines, positivity rates are about 13–15%,^5^ ^8^ in contrast with the 30–35% positivity rates reported in studies based exclusively on cytokeratin positivity without morphological analysis.^1^ Notably molecular analysis of cytokeratin-positive BM cells has shown that these cells may be malignant.^9^ ^10^ Clinical studies^11^ ^12^ have also demonstrated the prognostic value of cytokeratin-positive BM cells not classified as DTC and cytokeratin-positive cells.

In order to increase the number of epithelial cells found in the bone marrow, several workers have used immunomagnetic methods of selection (IMS). Available techniques use antibodies, linked to small paramagnetic beads or colloids of 1 nm (ferrofluids), with an affinity for specific cells. The cells can then be selected with a powerful magnet. Beads are available linked to antiepithelial antibodies for positive selection, like epithelial cell adhesion molecule (Ep-CAM), or linked to a monoclonal antibody directed against CD45 for negative selection of leucocytes.^13^ The use of negative IMS increased the frequency of positive BM in a large series from Norway but did not improve the prognostic value of this detection.^14^

Some studies have assessed DTC detection by using molecular biology techniques such as real-time quantitative PCR determination with several markers: CK19, MUC1,^15^ urokinase-type plasminogen activator receptor (uPAR), EpCAM^16^ and mammaglobin.^17^ This detection should be more sensitive (1 tumour cell in 10^7^ mononuclear cells) and more effective. However, using PCR to detect DTC raises two problems: firstly, the real problem of identifying appropriate sensitive and specific markers; and secondly, a problem of quantification. As no specific markers are available to detect DTC in BM, it is therefore not recommended to detect DTC in clinical trials by RT PCR alone without associated immunocytochemical detection.

### Prospects

DTC detection using more specific markers should improve the clinical relevance and reproducibility of this new parameter. One possibility would be to use markers that characterise each subtype of breast carcinoma, such as HER2, as this status is generally maintained in early metastasis such as DTC.^9^ ^18^ EGFR overexpression could also be promising in the basal-like subgroup.

## CLINICAL SIGNIFICANCE OF DTC

Several studies over the last two decades have assessed the prevalence and prognostic value of micrometastatic dissemination of breast cancer cells in bone marrow. These studies estimated that 12–45% of patients with primary operable breast cancer could have tumour cells in the bone marrow as determined by immunocytochemistry.^1^ ^5^ ^12^ ^19^^–^22 Table 1 summarises the major studies using immunocytochemical detection of DTC in BM. In a pooled analysis of nine studies comprising 4703 patients with stage I, II or III breast cancer, the presence of micrometastases at diagnosis was detected in 30.6% of patients. With a follow-up of 10 years, this analysis demonstrated that DTC in BM at the time of the initial diagnosis of breast cancer was a significant and independent prognostic factor with respect to poor overall survival and breast cancer-specific survival (univariate mortality ratios: 2.15 and 2.44, respectively; p<0.001 for both outcomes) and poor disease-free survival and distant disease-free survival during the 10-year observation period (incidence rate ratios: 2.13 and 2.33, respectively; p<0.001 for both outcomes). This evidence could be sufficient to include DTC analysis in the routine staging of primary breast cancer. However, technical issues remain controversial and bone marrow aspiration is not considered to be a convenient procedure for patients.

**Table 1:** Major clinical studies of the prognostic value of disseminated tumour cells (DTC) detection in bone marrow (BM) by immunocytochemistry and prognostic value on disease and overall survival (univariate and multivariate analysis)

Reference	Sampling	Marker	No. patients	Detection rate (%)	Follow-up (mth)	Disease free survival		Overall survival	
						Univ	Multiv	Univ	Multiv
Redding 1983^79^	Smear	MUC	110	28		NA	NA	NA	NA
Manegold 1988^80^	Biopsy	CK/PKK1	50	8		NA	NA	NA	NA
	Smear								
Landys 1998^81^	Biopsy	CK/AE1–AE3, KL1, CAM 5-2	128	19	240	NA	NA	Yes	NA
Salvadori 1990^82^	Biopsy	CK/MBr1	121	16.5	48	No	No	NA	NA
Mathieu 1990^83^	Biopsy	MUC/EMA, HMFG2	93	1		No	No	No	No
		CK/KL1, AE1–AE3, CAM5-2							
Kirk 1990^84^	Smear	MUC/anti-milk fat globulin LICR.LON.M8.4	25	48	34	No	NA	NA	NA
Singletary 1991^85^	Smear	CK/AE1, AE3, MAK-6	71	38	11	No	No	No	No
		MUC/113F1, 260F9, 317G5							
Cote 1991^86^	Smear	MUC/C26, T16	49	36.7	30	Yes	Yes	NA	Na
		CK/AE-1							
Schlimok 1992^87^	Cytospin	CK18/CK2	187	18	39	Yes	Yes	NA	NA
Harbeck 1994^88^	Smear	CK	100	38	34	Yes	Yes	No	Yes
		MUC/EMA							
Ménard 1994^89^	Cytospin	CK/MBr1, MBr8, CK18/CK2, MUC1	197	31	NA	NA	NA	NA	NA
Molino 1997^90^	Cytospin	CK/MBr1, MBr8, MOV8, MOV16 MluC1	109	31	36	No	No	No	No
Funke 1996^91^	Cytospin	CK18/CK2	234	38	NA	NA	NA	NA	NA
Diel 1996^92^,^93^	Smear	MUC/TAG12 (2E11)	727	43.3	78	Yes	Yes	Yes	Yes
Mansi 1999^12^,^94^	Smear	EMA	350	25.4	150	Yes	No	Yes	No
Lyda 2000^95^	Biopsy	CK/AE1–AE3, 35βH11 CAM 5-2	54	31	38	Yes	NA	NA	NA
Untch 1999^96^	Cytospin	CK18/CK2	581	28		No	No	No	No
Braun 2000^11^	Cytospin	CK/CK8,18,19 (A45 B/B3)	552	36	36	Yes	Yes	Yes	Yes
Gerber 2001^20^	Cytospin	CK/CK8,18,19 (5D3)	554	37	54	Yes	Yes	Yes	Yes
Gebauer2001^21^	Smear	CK, MUC/EMA	396	42	75	Yes	Yes	Yes	Yes
Kasimir-Bauer 2001^97^	Cytospin	CK/CK8,18,19 (A45 B/B3)	128	34	24	NA	NA	NA	NA
Naume 2004^5^	Cytospin	CK/AE1/AE3	819	13	49	Yes	Yes	Yes	Yes
Braun 2005^1^	Various	Various	4703	30.6	63	Yes	Yes	Yes	Yes
Bidard 2007^8^	Cytospin	CK/CK8,18,19 (A45 B/B3)	621	15	50	Yes	Yes	Yes	Yes

CK, cytokeratin; Muc, mucin; EMA, epithelial membrane antigen; NA, not available.

Peripheral blood would be an ideal source for the detection of tumoural cells, and sequential peripheral blood analyses are more acceptable. Depending on the detection technique used, circulating tumoural cells (CTC) were revealed in 50–100% of patients with metastatic breast cancer.^23^ Even in patients with no clinical signs of overt metastases, however, detection rates range from 10% to 60%.^24^ Detection of CTC with the CellSearch system (Veridex, Warren, New Jersey, USA), which detects CTC using Ep-CAM coated beads for enrichment followed pan CK staining, provided significant prognostic information before and also early (4 weeks) after initiation of chemotherapy in patients with metastatic breast cancer.^25^ CTC had superior and independent prognostic value of tumour burden and disease phenotype.^26^ In contrast to patients with metastatic disease, and despite promising results,^27^ the prognostic relevance of CTC in the blood of patients with early-stage disease without overt metastasis needs to be demonstrated in prospective multicenter studies.^28^

It is not clear if CTC measurements could replace the examination of bone marrow. Two immunocytochemical studies demonstrated statistically significant correlations between DTC detection in BM and CTC in blood, but BM was more frequently positive than blood.^29^ ^30^ Recently, Benoy *et al* found that real-time RT-PCR based detection of DTC in BM had superior significance to CTC measurements in blood.^31^ In addition, Wiedswang *et al*, with an ICC assay, showed that BM but not blood analyses provided prognostic information.^32^ These finding do not support an exchange of DTC in BM with CTC from blood.

An important potential application for DTC detection is the monitoring of therapeutic efficacy in the adjuvant setting which can currently only be assessed retrospectively in large-scale clinical trials after an observation period of at least 5 years. Persistence of DTC in BM some years after diagnosis and initial therapy is still an indicator of subsequent systemic treatment failure.^33^^–^35 Persistence or disappearance of DTC after systemic treatment could therefore be used as a surrogate marker of treatment response.^36^ Studies have shown that adjuvant chemotherapy has no effect on the elimination of single dormant tumour cells in the BM of high-risk breast cancer patients.^37^ ^38^ This emphasises the need to develop therapeutic agents that are active on non-proliferating cells. Bisphosphonates have been used to eliminate tumour cells in BM persisting after adjuvant therapy. The most promising agents are antibodies such as edrecolomab directed against EpCAM^39^ or trastuzumab directed against HER2.^40^ Large-scale prospective clinical trials must now be conducted to determine whether eradication of DTC in BM after systemic therapy results in longer survival.

## BIOLOGICAL AND CLINICAL QUESTIONS RAISED BY MICROMETASTATIC CELLS

The micrometastasis phenomenon is usually described as “tumour cell dormancy”,^41^ added as a late step of the metastatic cascade.^42^ Although dormancy regulation is a key element of micrometastasis biology, micrometastatic cells can be assumed to be more than just dormant cancer cells; they could help us to understand certain aspects of metastasis biology. The main clinical and biological questions raised by the micrometastatic process and the current answers to these questions, are described below.

### How and when does micrometastasis occur?

The main clinical study reported that cytokeratin or mucin positive BM cells are associated with tumour size, grade, negative hormone receptors and lymph node metastases.^1^ Using a more stringent detection technique, we did not reproduce any of these results,^8^ although they have been confirmed by others.^43^ This might suggest that micrometastatic dissemination occurs in highly proliferative tumours, when a critical tumour size has been reached. However, DTC may be found at earlier stages of primary tumour development, and comparative genomic hybridisation analyses of disseminated cancer cells were in favour of early dissemination of breast cancer cells to the BM.^10^ ^44^  Supervised transcriptomic profiling of 19 primary tumours has been reported,^45^ but this micrometastasis-associated profile has not been further validated by independent unsupervised analysis. No pathological studies have demonstrated a link between micrometastasis detection and the recently described breast cancer subtypes.^46^ Therefore, although BM DTC may appear early, it is unknown whether they correspond to a genetically homogeneous subgroup of primary cancers.

Among the mechanisms of breast cancer cell dissemination to the BM, bone and bone marrow homing of cancer cells may depend on similar molecular determinants, especially the SDF1/CXCR4 axis.^47^ ^48^ CXCR4 is a G protein-coupled receptor,^49^ which plays a role in the chemotaxis of breast cancer cells. This cellular response is attributed to activation of the PI3K/PTEN/AKT/mTOR signalling pathway,^49^ rather than the MAP/ERK pathway.^50^ ^51^ CXCR4 expression in 142 primary breast cancers has been shown to be associated with the detection of BM DTC.^52^ This pathway might be responsible for early dissemination of breast cancers, as circulating cancer cells detected in the blood are also characterised by activation of PI3K.^53^ Epithelial-mesenchymal transition and primary tumour microvessel density may also be involved in the onset of DTC.^54^ ^55^ Finally, the molecular determinants responsible for the establishment of BM DTC are not clearly understood at the present time. BM DTC could be a useful tool to assess the efficiency of the entire cancer cell migration process and should be analysed together with circulating tumour cells in blood and primary breast tumours.

### Are micrometastatic cells metastatic progenitors?

Bone marrow is the host organ of breast cancer metastases, which is the most accessible tissue for analysis, as liquid aspirates can be performed under local anaesthesia or during primary surgery. It is of critical importance to determine whether DTC are (or are not) the metastatic progenitors of bone and/or distant non-bone metastasis. A negative answer would limit the accuracy of bone marrow DTC as a biological model, a target for adjuvant treatment and a marker of response. Paget^56^ was the first to describe the non-random growth of metastases, and the sustaining molecular determinants of cancer cell homing have been recently characterised.^52^ ^57^ ^58^ If BM DTC are derived from the specific spread of a few tumour subclones into flat bones, their ability to recirculate to other organs would be somehow limited. Consequently, their detection would be linked to an increase of bone metastasis at primary relapse in patients, but not to that of other metastatic sites. Micrometastases retained their clonogenic and tumourigenic capacities in many biological reports.^59^^–^61 Clinical studies have reported a link between BM DTC and the onset of bone metastasis,^1^ ^8^ ^43^ strongly supporting the idea of local growth of DTC into macrometastases.

Surprisingly, other organs (especially the liver) also appear to be a favourite site of breast cancer relapse.^8^ ^43^ However, the CXCR4 receptor is reported to be involved in homing to both sites,^52^ but the current literature does not provide strong evidence for a common pool of genes responsible for coupled homing to bone marrow (or flat bones) and liver. The other alternative is that bone marrow may act as a long-term reservoir of tumour cells, which can recirculate to other distant organs before growing into metastases.^62^ The high genetic heterogeneity^63^ of BM micrometastatic cells might be responsible for recirculation of some cancer seeds from the bone marrow to different host organs. However, no biological or clinical study has directly reported such a process for BM DTC and there is currently no direct evidence suggesting that they are responsible for the late growth of lung of liver metastases. On the contrary, many biological models have reported that the micrometastatic dissemination of mammary tumours occurs in most of the target organs of metastases^64^^–^66 and is not restricted to a unique reservoir in the bone marrow. Also, 40 months after the surgical treatment of non-metastatic breast cancer, the detection of circulating cancer cells in the peripheral blood was not correlated with the presence of bone marrow DTC.^32^ Finally, the local growth of some BM DTC into bone macrometastasis is clinically and biologically rational. In the case of distant non-bone or local relapses predicted by BM DTC,^8^ these cells mostly appear as a marker of a body-wide dissemination of invasive cancer cells rather than the body’s only long-term reservoir of disseminated cancer cells.

### How is micrometastatic dormancy regulated?

Dormancy may be induced in disseminated cancer cells by lack of the primary tumour microenvironment (absence of stimulating growth factors,^67^ presence of growth-inhibiting cytokine^68^). Metastatic growth may be a rare and stochastic event secondary to selection and mutations of dormant cancer cells.^69^ The end of dormancy may also be induced by any change of the microenvironmental homoeostasis, such as the presence of growth factors or an immune response.^70^ Many groups investigating the mechanisms of dormancy have reported the role of integrin α5 β1 in regulating breast cancer cell dormancy. This integrin is activated by the urokinase-type plasminogen activator receptor (uPAR). Its main signalling pathway is the FAK/Src and ERK pathway to promote cell mobility; inhibition of this integrin leads to cancer cell dormancy in biological models.^71^ ^72^ Integrin α5 β1 appears to be necessary, via the PI3K/AKT pathway, for the survival of dormant cancer cells.^68^ uPAR expression by BM DTC has also been linked to a poorer prognosis in a population of micrometastatic patients.^16^

Regulation of cancer cell dormancy may also involve genes and other processes, which regulate primary tumour growth. Finally, most BM DTC-positive patients never relapse, while others experience dramatic metastatic progression. These different outcomes cannot be explained at the present time and require further investigation.

### Micrometastasis and cancer stem cells

The role of cancer stem cells in the establishment of metastasis remains controversial in many theoretical proposals and reviews.^73^^–^76 Experimentally, CD44+/CD24−/low cancer cells, a phenotype associated with a stem cell pattern, exhibit an invasive phenotype,^77^ ^78^ which is a prerequisite to metastasis. In a report on 50 cases, most BM micrometastatic breast cancer cells exhibited a stem cell-like immunohistochemical phenotype.^76^

## CONCLUSION

BM DTC detection is a very promising prognostic parameter that will improve clinical management of patients with breast cancer in the near future. DTC detection must now be implemented in clinical trials to improve treatment selection. DTC specificity is considerably increased by histological examination according to international guidelines and must be submitted to high-level quality control. The future development of targeted therapies against BM DTC should significantly improve patient outcome and raises interesting new biological questions that should further our understanding of breast cancer carcinogenesis.

**Take-home messages**

- Immunocytochemical detection of disseminated tumour cells in the bone marrow of primary breast cancer patients at surgery is an independent prognostic factor of poor outcome.
- Accurate identification of disseminated tumour cells (DTC) in bone marrow must be based on standardised methodologies and procedures.
- Prognostic value of circulating tumour cells (CTC) detection in peripheral blood is demonstrated in patients with metastatic disease but remains to be substantiated at early stage.
- DTC and CTC detection should be used in stratification and monitoring of therapeutic efficacy.
- Research on DTC could help in understanding tumour dormancy and identifying metastatic stem cells.
